# A rat model of radiation injury in the mandibular area

**DOI:** 10.1186/s13014-015-0432-6

**Published:** 2015-06-09

**Authors:** Tonje Sønstevold, Anne Christine Johannessen, Linda Stuhr

**Affiliations:** Department of Biomedicine, Faculty of Medicine and Dentistry University of Bergen, Serviceboks 7804, N-5020 Bergen, Norway; The Gade Laboratory for Pathology, Department of Clinical Medicine, Faculty of Medicine and Dentistry, University of Bergen, Bergen, Norway

**Keywords:** Radiation injury, Head and neck cancer, Animal model, Mandible, Salivation, Vascularity, Collagen, Tooth development

## Abstract

**Background:**

Radiation technology focuses on delivering the radiation as precisely as possible to the tumor, nonetheless both acute and long-term damage to surrounding normal tissue may develop. Injuries to the surrounding normal tissue after radiotherapy of head and neck cancer are difficult to manage. An animal model is needed to elucidate good treatment modalities. The aim of this study was to establish a rat model where a certain radiation dose gives reproducible tissue reactions in the mandibular area corresponding to injuries obtained in humans.

**Method:**

The left mandible of male Sprague Dawley rats was irradiated by external radiotherapy (single fraction 15 Gy, total dose 75 Gy) every second week five times. Endpoint was six weeks after last radiation treatment, and the test group was compared to non-irradiated controls. Morphological alterations of the soft tissues, bone and tooth formation, as well as alterations of salivation, vascularity and collagen content were assessed. An unpaired, non-parametric Mann–Whitney test was used to compare the statistical differences between the groups.

**Results:**

Analysis of the soft tissues and mandible within the radiation field revealed severe unilateral alopecia and dermatitis of the skin, extensive inflammation of the submandibular gland with loss of serous secretory cells, hyperkeratinization and dense connective fiber bundles of the gingival tissue, and disturbed tooth development with necrosis of the pulp. Production of saliva and the vascularity of the soft tissues were significantly reduced. Furthermore, the collagen fibril diameter was larger and the collagen network denser compared to non-irradiated control rats.

**Conclusion:**

We have established an animal model of radiation injury demonstrating physiological and histological changes corresponding to human radiation injuries, which can be used for future therapeutic evaluations.

## Background

Head and neck cancer (HNC) is a heterogeneous group of neoplasms that share a common anatomic origin. These tumors develop within the mucosa that lines the upper aerodigestive tract (squamous-cell carcinomas) or the different glands in this region (adenocarcinomas). Thus, HNC includes carcinomas of the salivary glands, oral cavity, nasal cavity, lip, pharynx and larynx [1, 2]. Treatments of HNC include surgery, radiotherapy, chemotherapy or targeted therapy, administered either alone or in combination [3].

The majority of HNC patients need radiotherapy at one point during treatment. The ionizing radiation interrupts the growth of cancerous cells by causing direct damage to the DNA and other cell components, or through the formation of free radicals. Cells exhibit different levels of radiosensitivity depending on their stage in the cell cycle at the time of radiation [4]. Tissues containing rapidly dividing cells are highly sensitive to radiation and therefore termed early-responding tissues, while tissues with slower turnover rates are less sensitive and are thus called late-responding tissues. Normal cells have a greater capacity than tumor cells to repair the radiation damage, especially at low doses [5]. This makes fractionated radiotherapy efficient in sparing normal tissue. Most patients with HNC, treated with curative intent, receive a total dose between 50 and 70 gray (Gy), where the dose is parceled into fractions, usually 2 Gy daily, five days a week over a five to seven week period [5]. Nevertheless, radiation injuries do occur in normal tissue and are commonly classified as acute, consequential or late complications. Acute effects are observed during or within a few weeks after treatment. Consequential effects are defined as persistent acute damage. Late effects are, on the other hand, typically seen after a latent period of six months or more, and may occasionally develop years after exposure to radiation [6, 7]. The late radiation injuries largely reflect damage to vascular and connective tissue, reducing vascularity and increasing fibrosis, which may finally result in cell death [6]. In its most severe form late radiation injuries of the head and neck can progress to cutaneous fistulas, trismus, pathologic fractures and osteoradionecrosis [8]. This severe form of radiation injuries are often precipitated by an additional tissue insult, such as surgery within the radiation field [9]. As it would be unethical to expose the animals to such a degree of radiation damage, a model developing the most severe forms of radiation injuries is not the scope of this study and thus not discussed in further detail.

Limiting the radiation dose and dose rate are the primary ways of preventing complications. Conventional treatment of radiation injuries in the head and neck region today involve antiseptic oral solution and better oral hygiene, anti-inflammatory drugs and parenteral antibiotics. Additional treatment can be stimulation of the residual secretory capacity of the salivary glands or the use of saliva replacements, debridement or sequestrectomy, and anesthetics and analgesics for pain relief [10]. The lack of a representative animal model for radiation injuries of the head and neck region in general has made studies on possible treatment modalities difficult. Consequently, establishing a reproducible and reliable animal model for such studies was the aim of the present study.

## Materials and methods

### Animals

Adult male Sprague Dawley rats (Taconic Farms, Inc, Denmark) with an average body weight of 350 g were used in this study. The rats were housed in individually ventilated cages (IVC type IV, Techniplast, Italy), fed standard pellets (RM1, Special Diets Services, Scanbur A/S, England) and had access to both food and water *ad libitum*. They were randomly divided in two groups: controls and test rats (15 Gy every other week five times). The rats were carefully monitored, recording skin changes, hair loss, eating ability, overall activity, clouding of eyes, wounds or infections and body weight (less than 20 % weight loss was set as acceptable). During the last period of the experiments the pellets were soaked in water to ease the eating process due to the side effects of radiation, such as dryness and soreness of the mucosal linings in the mouth. Their incisors were cut to prevent them from growing too long. The study was approved by the local ethical committee (project number 20113900), and all experiments were performed in accordance with the recommendations of the Norwegian State Commission for Laboratory Animals.

### Radiation treatment

The radiation was performed under isoflurane (Isoba®vet. 100 %, Schering-Plough A/S, Denmark) and N_2_O inhalation anesthesia at Haukeland University Hospital, Department of Radiophysics, using a Varian Linear Accelerator, Clinac 600C/D (Reg.no. 26830).

The radiation regime was selected on the basis of preliminary studies testing single doses of 30–60 Gy targeted to the left mandible, and pilot studies testing fractionation schemes of 4 × 15 Gy, 6 × 10 Gy and 5 × 15 Gy targeted to the left mandible every other week. As a result of the pilot studies the present study employed the 5 × 15 Gy fractionation scheme, distributing 15 Gy every other week five times, cumulating to a total dose of 75 Gy. The animals were sacrificed six weeks after the final radiation treatment.

**Fig. 1:**
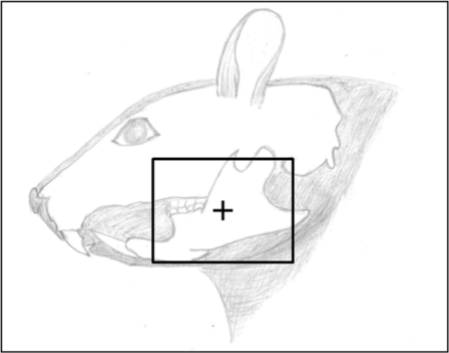
Schematic presentation of the radiation field. The box indicates the area of the head and neck region irradiated, and the cross indicates where the isocenter of radiation is targeted to the mandible

The animals were positioned on their right side, so as to irradiate the left mandible. Palpation and laser light was used as the method of locating the isocenter, which was the mandibular body between the angle process and the molars as shown in Fig. 1. The collimator was set to a radiation field of 2.6 × 3.5 cm at a distance of 75 cm from the source. A 10 mm bolus was applied above the mandible to compensate for dose depth. The linear accelerator was set to give the specific dose of 1024 motor units (MU) equal to 15 Gy, and the rats were irradiated with 6 MV gamma rays at a dose rate of 400 MU/min. Any unwanted radiation outside the targeted mandibular area was prevented using lead blocks positioned at the edge of the radiation field, by help of polystyrene on each side of the rat and a Plexiglas on top.

### Salivation

Saliva measurements were performed under anesthesia (i.p.) with ketamine at a dose of 80 mg/kg (Ketalar, Pfizer AS, Norway) and medetomidine at a dose of 0.5 mg/kg (Domitor®vet., Orion Pharma, Finland). Saliva production was stimulated by using the parasympathomimetic agent Pilocarpine (1 μg/g BW) (Pilocarpinehydroklorid, Sigma-Aldrich Norway AS) and collected for 15 min with pre-weighed Sugi sponges on each side of the tongue from the first sign of increased salivation. The procedure was performed under a heating lamp, and the head of the rat was kept lower than its body. The salivation measure was based on the weight difference of the Sugi sponges with and without saliva absorption.

### Dissection and histology

The rats were sacrificed after the saliva measurements by intracardiac injection of saturated KCl under anesthesia. The skin, muscle and submandibular gland (left) within the radiation field were gently dissected out. All soft tissue samples were either fixed in 4 % paraformaldehyde (PFA) before embedding in paraffin for subsequent histological analysis, or snap frozen in liquid nitrogen and stored at −80 °C for subsequent immunostaining. The left part of the mandible was dissected out, cleaned of detritus and cut in front of the last molar in two parts (front and back). All mandibles were fixed in 4 % PFA, for at least four hours, before they were decalcified in 10 % ethylenediaminetetraacetic acid (EDTA) solution, pH 7.4, for five weeks. The mandibles were then either dehydrated in 70 % ethanol before embedding in paraffin, or washed in 0.1 M phosphate buffer, pH 7.4, and placed in 30 % sucrose overnight and further stored at −80 °C.

The paraffin embedded tissue was sectioned in 4–5 μm sections and stained with Hematoxylin and Eosin (H&E) for tissue morphology. The frozen tissue specimens were sectioned in 10 μm sections and used for immunohistochemistry to label endothelial cells, more specifically rat platelet endothelial cell adhesion molecule-1. The frozen tissue sections were fixed in acetone for 10 min and washed in phosphate buffered saline (PBS), pH 7.4, between every step unless otherwise stated. Endogenous peroxidase activity was blocked by 30 min incubation with 0.3 % H_2_O_2_ in methanol. Unspecific binding sites were blocked by 20 min incubation with normal rabbit serum. Excess serum was tapped off and sections were incubated with primary antibody Monoclonal Mouse anti-Rat CD31, clone TLD-3A12, (MCA1334G, AbD-Serotec, Oxford, 1.0 mg/ml) diluted 1:50 in PBS with 0.3 % Triton® X-100 (PBS-TX), pH 7.4, for 60 min at room temperature. Further, sections were incubated with secondary antibody Biotinylated Polyclonal Rabbit anti-Mouse Ig G (E0464, DakoCytomation, Denmark) diluted 1:200 in PBS-TX for 30 min. The ABC Reagent (Peroxidase Rat IgG, PK-4004, Vectastain ABC-kit, Vector Laboratories, Burlingame) was applied and incubated for 30 min. Substrate-Chromogen from Dako EnVision + System-HRP (DAB+) kit (K4006, DakoCytomation, Denmark) was applied and incubation was performed until the desired stain intensity had developed. Sections were rinsed continually for approximately 10 min. Counterstaining was performed with Richardson’s stain, and sections were subsequently washed in dH_2_O and dehydrated in increasing concentrations of ethanol (50 %, 75 %, 96 % and 100 %) and then xylol. Sections were mounted with Histokitt and cover-slipped. Specimens were examined with a light microscope (Nikon Eclipse E600, 724064, Japan) connected to a camera (Nikon Digital Sight DS-U3, 250430, Japan) and a computer screen. The IHC stained sections were analyzed manually and the average CD31 density per mm^2^ calculated for the control and the irradiated group (n = 5 rats).

### Transmission electron microscopy

To study the difference in collagen density of irradiated versus non-irradiated skin tissue, samples from the outer part of the radiation field were prepared for transmission electron microscopy, as previously described [11]. The sections were studied by use of a transmission electron microscope (JEM-1230, Jeol, Japan) connected to a GATAN multiscan camera. The collagen fibril density was manually evaluated. Five representative images from different areas were obtained per rat (40 000X objective), and the average collagen density was calculated per μm^2^ (n = 5 rats) for the control and the irradiated group. The collagen fibril diameter was measured using ImageJ. The diameter was measured on approximated circle round cross-sectioned fibrils at the shortest distance across the fibril. Twenty cross-sectioned fibrils from five different areas of the skin sample were measured per rat (80 000X objective, n = 5). The fibrils were further clustered according to diameter to present the frequency distribution.

### Statistics

An unpaired, non-parametric Mann–Whitney test was used to compare the statistical differences between the two groups. A value of *p* < 0.05 was considered statistically significant.

## Results

To determine the impact of 5 × 15 Gy radiotherapy targeted to the left mandible parameters such as histology, salivation, vascular density and collagen content were analyzed in the skin, muscle, submandibular gland and mandible of the radiation field. All rats survived the radiation period of 14 weeks.

### Clinical findings

All the irradiated animals demonstrated severe unilateral left cheek skin alopecia and mild dermatitis when compared to the control group. The hair loss in the field of radiation started following the third treatment and was potentiated following the last treatment. It was evident that the skin exposed to radiation was irritated, and this coincided with an increased scratching behavior. As the skin in the radiation area became hairless, other responses to the radiation became more visible such as dry desquamation, pigmentation changes, ulceration and increased roughness. Upon dissection, the jaw region exposed to radiation generally demonstrated hard, tough tissue linings and extensive connective tissue growth that was not observed in controls.

### Salivation

**Fig. 2:**
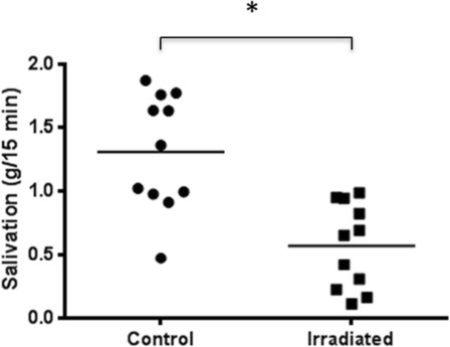
Salivation six weeks after 5 × 15 Gy radiotherapy. Salivation measured as grams saliva per 15 min in control (*n* = 11) and irradiated (*n* = 11) rats. The horizontal line indicates the mean values. *indicates *p =* 0.0004 by an unpaired, non-parametric Mann–Whitney test

To investigate the salivary glands’ ability to produce saliva after radiotherapy, salivation was measured using Pilocarpine. The average salivation (mean ± SD) was measured to be 1.31 ± 0.46 g saliva per 15 min in the control rats and this was significantly reduced to 0.57 ± 0.34 g saliva per 15 min (*p <* 0.001) in irradiated rats (Fig. 2). These results are consistent with the tissue changes described below for the salivary glands.

### Histology

**Fig. 3:**
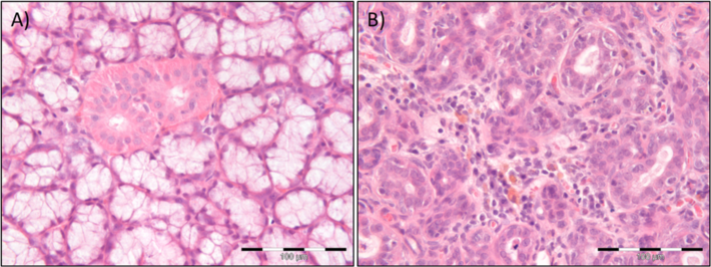
Changes in salivary gland morphology six weeks after 5 × 15 Gy radiotherapy. **a** H&E stained section of the left submandibular gland of non-irradiated control rats. The histologic examination primarily demonstrated acini and excretory ducts without any inflammatory reactions. Scale bar indicates 100 μm. **b** H&E stained section of the left submandibular gland of 5 × 15 Gy irradiated rats. The histologic examination revealed atrophy of the acini, dilated excretory ducts and blood vessels, hemosiderin and chronic inflammation. Scale bar indicates 100 μm

The salivary gland morphology was macroscopically affected by the radiation in approximately half of the irradiated animals, being either enlarged or pale compared to the non-irradiated control rats. The H&E stained sections of the submandibular gland from both control and irradiated animals were examined. The control gland showed acini with serous secretory cells, normal excretory ducts and blood vessels (Fig. 3a). The irradiated gland showed atrophy of the acini with loss of serous cells, more fibrous tissue, dilated blood vessels and excretory ducts, hemosiderin (signs of prior tissue bleeding) and an ongoing chronic inflammation with abundant mononuclear cells (Fig. 3b).

**Fig. 4:**
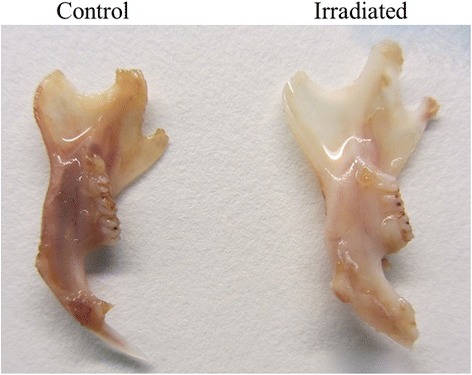
Changes in mandibular morphology six weeks after 5 × 15 Gy radiotherapy. The non-irradiated control mandible demonstrated darker coloration (left) after five weeks of decalcification with 10 % EDTA compared to the irradiated mandible (right)

**Fig. 5:**
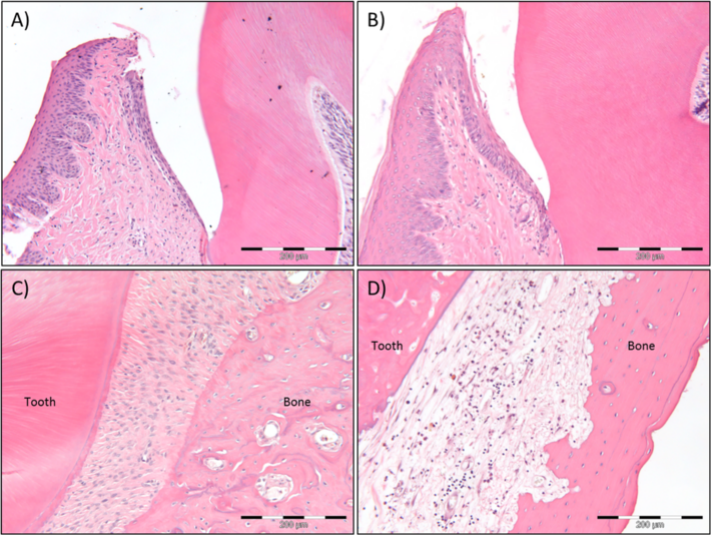
Morphologic changes of gingiva and periodontal membrane six weeks after 5 × 15 Gy radiotherapy. H&E stained sections of the gingiva of non-irradiated control rats (**a**) and 5 × 15 Gy irradiated rats (**b**). The histologic examination demonstrates epithelial transformation from slightly parakeratinized in controls to hyperkeratinized and hyperplasic in irradiated rats. This is also evident in the epithelium closest to the tooth. In addition the connective tissue is more fibrous and contains fewer cells after irradiation. Scale bar indicates 200 μm. (**c**) H&E stained sections of the periodontal membrane of non-irradiated control rats. The periodontal membrane is richly vascularized with fibers organized for proper attachment of tooth to bone. Scale bar indicates 200 μm. (**d**) H&E stained sections of the periodontal membrane of 5 x15 Gy irradiated rats. The irradiated periodontal membrane demonstrates fibrous connective tissue with few cells, unorganized fibers and infiltration of mononuclear inflammatory cells. Scale bar indicates 200 μm

**Fig. 6:**
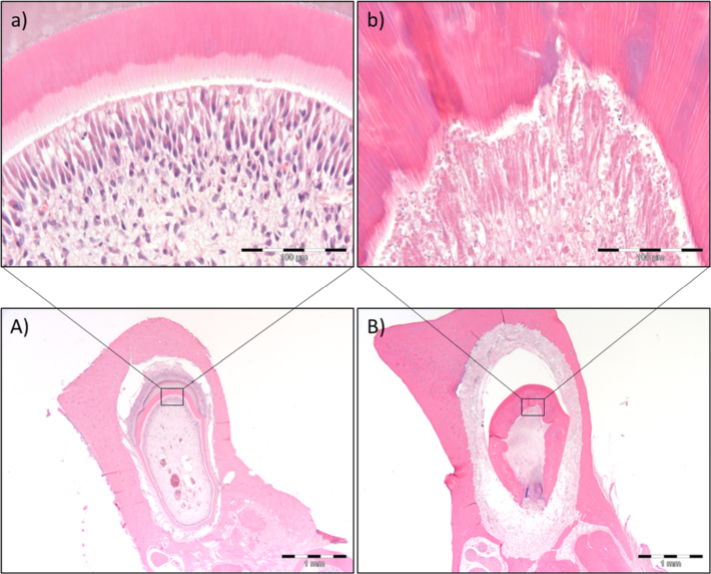
Morphologic changes of teeth under development six weeks after 5 × 15 Gy radiotherapy. H&E stained sections of developing teeth of non-irradiated control rats (**A, a**) and 5 × 15 Gy irradiated rats (**B, b**). The histologic examination demonstrates disturbed enamel, dentin and cementum formation after irradiation, as seen from the overview section **B**) compared to **A**). Scale bar indicates 1 mm. Furthermore, disturbed and unorganized dentin formation and necrosis of odontoblasts and pulp was evident after irradiation, as seen from the close up section **b**) compared to **a**). Scale bar indicates 100 μm

During fixation with PFA and subsequent decalcification with 10 % EDTA, the irradiated mandibles demonstrated a considerable paler color than the non-irradiated control mandibles (Fig. 4). Histologically, the gingival tissue showed hyperkeratinization, not only in the oral gingival epithelium, but also in the pocket epithelium. All connective tissue related to the tooth, i.e., gingival tissue and periodontal membrane, showed dense connective fiber bundles and a reduced number of fibroblasts, which also were more spindle shaped, compared to control tissue (Fig. 5). Additionally there were fewer vessels, but these had a larger diameter. Teeth under eruption showed disturbed root development, while the crown was seen to be normal. In unerupted teeth completely disturbed tooth development was observed, with disturbed dentin- and cementum formation, pulp necrosis, and even acute inflammation (Fig. 6). The bone was vital, but no osteoblasts could be identified.

### Vascular density

**Fig. 7:**
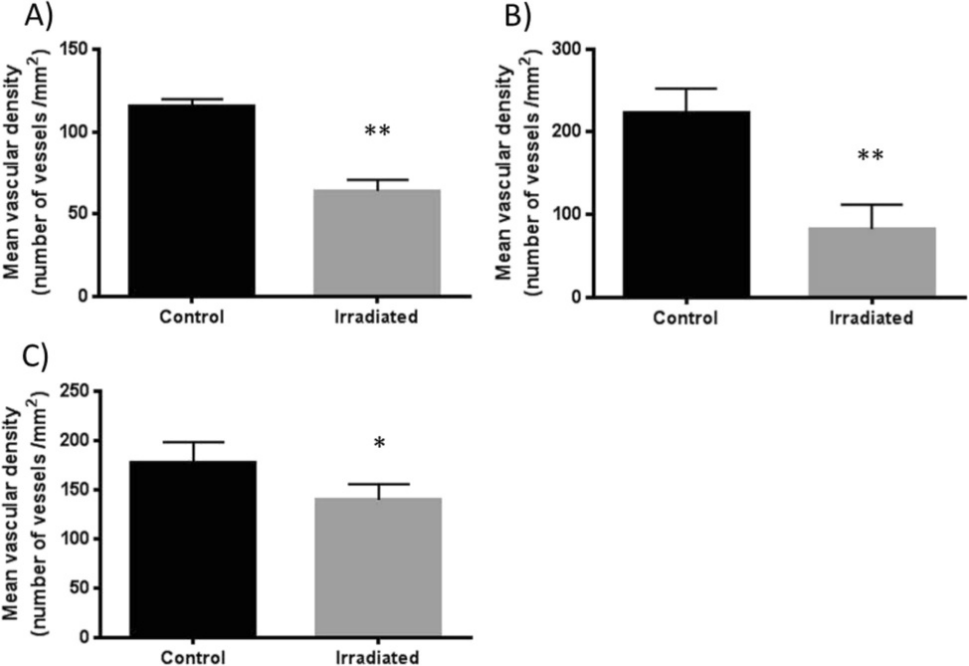
Vascular density six weeks after 5 × 15 Gy radiotherapy. The average vascular density by immunohistochemistry with CD31 in the skin (**a**), the muscle (**b**) and the submandibular gland (**c**) of control (*n =* 5) and irradiated (*n =* 5) animals presented as mean ± SD. *indicates *p* < 0.02 and **indicates *p* < 0.01 by an unpaired, non-parametric Mann–Whitney test

The effect of radiation on the vasculature of the different tissues within the radiation field was of particular interest. The number of CD31 positive blood vessels per mm^2^ was significantly reduced in the skin (*p* < 0.01), the masticatory muscle (*p* < 0.01) and submandibular gland (*p* < 0.02) six weeks after 5 × 15 Gy radiotherapy compared to non-irradiated controls (Fig. 7).

### Collagen density of the skin

**Fig. 8:**
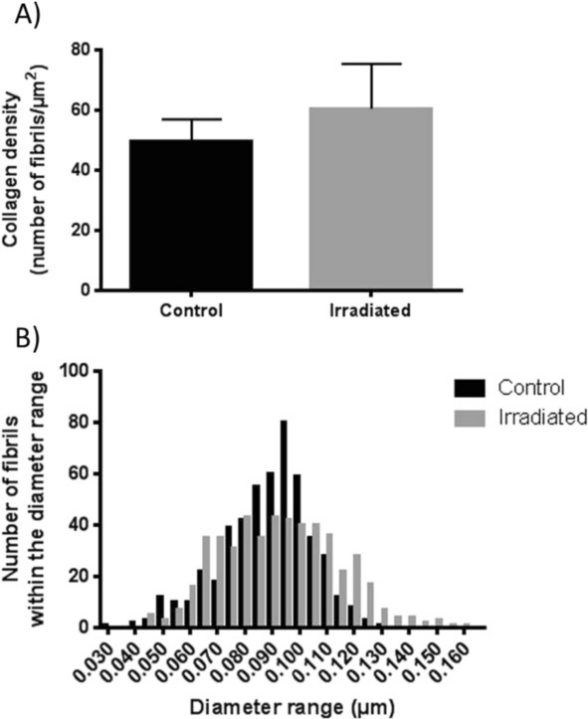
Collagen density and diameter six weeks after 5 **×** 15 Gy radiotherapy. (**a**) The average fibril density in control (*n =* 5) and irradiated (*n =* 5) skin samples from the outer part of the radiation field presented as mean ± SD. (**b**) The frequency distribution of collagen fibril diameter demonstrates a minor displacement towards larger fibril diameter in the skin after radiotherapy (*n =* 5) compared to control animals (*n =* 5)

**Fig. 9:**
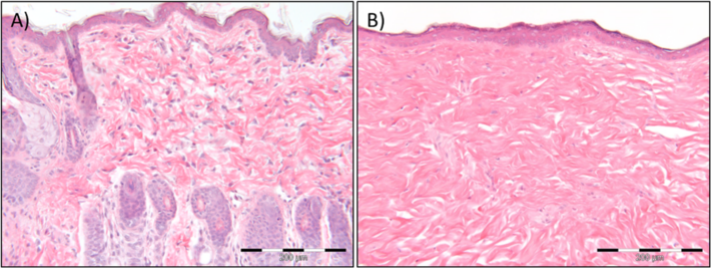
Changes in skin morphology six weeks after 5 × 15 Gy radiotherapy. H&E stained section of the skin epidermis and dermis of non-irradiated control rats (**a**) and 5 × 15 Gy irradiated rats (**b**). The histologic examination demonstrates alopecia and a dense network of collagen fibers with few cells, increased fibrosis, of the dermis after irradiation. Furthermore, the stratum corneum, the outermost layer of the epidermis, is thicker after irradiation. Scale bar indicates 200 μm

Collagen fibril density was measured to determine the degree of fibrosis after radiotherapy. No statistically significant increase of collagen fibrils was evident in skin samples taken from the outer part of the radiation field six weeks after 5 × 15 Gy radiotherapy (Fig. 8a), although there seemed to be a trend towards an increased fibril density. However, fibrosis was clearly verified in the H&E stained histological sections taken from the isocenter of radiation (Fig. 9). The average fibril diameter of the irradiated group was 0.093 μm, while for the control group it was 0.088 μm. The fibrils were grouped according to their diameters to demonstrate the frequency distribution, as shown in Fig. 8b. The results showed a minor displacement towards larger collagen fibril diameter six weeks after 5 × 15 Gy radiotherapy compared to controls.

## Discussion

The present study aimed to establish an animal model of radiation injuries corresponding to injuries of patients receiving radiotherapy as treatment for HNC. However, the study did not aim to produce the more severe forms of late radiation injuries. A linear accelerator was utilized, and a cumulative radiation dose of 75 Gy, fractionated in 15 Gy treatments distributed every second week five times, with a six week post-radiation period was found to induce reproducible tissue reactions in the mandibular area, similar to what is obtained in humans.

### Pilot studies

Previous animal studies, using single doses between 20 and 50 Gy distributed alone or together with tooth extraction or distraction, have reported an induction of radiation damage [12–17]. In preliminary studies conducted in our laboratory, the dose-survival response with single doses of 30–60 Gy was tested. The animals exposed to 30 and 60 Gy were sacrificed prior to study termination due to severe health effects. The animals exposed to 50 Gy did, however, survive the post-radiation period of six weeks, but considering the well-being of the animals and the clinical relevance of the experiments, fractionated therapy was chosen for the next step of the study. A study on rats by Fenner et al. [18] demonstrated radiogenic bone damage following external irradiation with a fractionation scheme of 4 × 15 Gy obtaining a cumulative total dose of 60 Gy [18]. Consequently, this was tested during the pilot studies for our animal model. However, minor histological changes attributable to radiotherapy were registered when this was tested in our laboratory. With regards to the preliminary studies, and to keep within clinically relevant doses for treatment of humans, it was decided to further test 5 × 15 Gy and 6 × 10 Gy, obtaining a total reference dose of 75 Gy and 60 Gy respectively.

The pilot study showed the 6 × 10 Gy protocol only produced minor tissue reactions compared to the 5 × 15 Gy protocol. This is consistent with a study by Thames et al. [19], who found that the dose per fraction is of greater importance for the causation of late injuries than the actual number of fractions. Early-responding tissues and late-responding tissues exhibit different dose-survival curves. For early-responding tissues, a low dose given multiple times weekly gives equivalent acute injuries to larger doses given fewer times a week, generating the same total radiation dose. However, when increasing the dose per fraction and reducing the number of fractions (obtaining the same total dose) this results in increased injury in late-responding tissues. Consequently, a significant worsening of late effects would accompany an increase in dose per fraction, with no change in the span of acute responses [19]. Correspondingly, the 10 Gy fractions did not render enough damage to establish a radiation injury of the quality sought in the present animal model.

In contrast to our radiation protocol, the fractionation schemes applied to humans employ 2 Gy daily, five days a week for five to seven weeks. This would not be applicable in our experiment, since the procedure would be too cumbersome and costly. In general, the severity of tissue damage increases with total dose and fraction size. Thus, the use of multiple, small radiation fractions rather than one or a few large fractions decrease late effects on normal tissue [4, 19].

A latent period of six weeks after radiation was employed as the endpoint of choice in the present study because rodents are known to have a metabolic rate four to six times higher than in humans. The post-radiation interval would thus be comparable to a follow-up period of 24–36 weeks (approximately six months) in a patient situation [18, 20], equal to the common latent period of late radiation complications [7]. This choice was strengthened in a study by Fenner et al. [18], who demonstrated no major supplementary findings in their twelve weeks follow-up compared to the one of six weeks [18]. Further, to confirm that the radiation injuries of the present animal model were persistent and non-healing a follow-up study was performed with latent periods of 8, 10 and 12 weeks. All latent periods produced the same radiation injuries as the 6 week latent period.

### Morphologic changes of the skin

During the course of radiotherapy in this animal model, dermatitis was observed. The skin became inflamed and irritated, and the rats showed increased scratching behavior. Dermatitis and mucositis are among the most common symptoms reported following external radiotherapy for humans [6]. The rapid response of the skin when exposed to radiation coincides with the skin’s high abundance of proliferating cells, making the skin highly radiosensitive [4]. In our animal model, the acute symptoms were potentiated when the radiation treatments ended and a total radiation dose of 75 Gy had been distributed. This correlates with reports from human studies where late radiation changes rarely occur until doses greater than 50 Gy are imposed [21]. Skin alopecia, pigmentation changes, dry desquamation and rough skin were demonstrated in the radiation field of all animals six weeks after radiotherapy. This is consistent with human observations. Repeated exposure to high doses of radiation does not allow time for the damage to be fully repaired, and hence the self-renewing property of the epidermis gets disrupted [22]. The potentiation of the acute symptoms into the late phase are caused by progressing vascular damage, as also found in the present study, in addition to fibrosis leading to edema, induration and thickening of the dermis, atrophy and necrosis [6, 21, 22].

### Salivation and morphologic changes of the salivary gland

Salivary gland dysfunction in the present animal model was confirmed by salivation measurements using pilocarpine. The saliva production was significantly reduced six weeks after 5 × 15 Gy radiotherapy. Xerostomia is a frequently occurring side effect seen in patients receiving radiotherapy as HNC treatment [21]. Severe exterior morphologic alterations of the submandibular gland were observed in approximately half of the irradiated animals. The histological analyses verified chronic inflammation, reduced number of secretory cells, more fibrous tissue and both dilated blood vessels and excretory ducts after radiation in our animal model. This is in agreement with the histopathological changes observed in humans following fractionated radiotherapy with total doses of 50–70 Gy [21]. Normal saliva secretion could not be maintained as there were fewer secretory cells. The resultant scant and sticky saliva may cause subsequent dental caries and infections, worsening the radiation damage [5]. From the existing human and animal studies it appears that radiation damage of the salivary glands occurs as a direct effect on secretory cells and ducts rather than being secondary to vascular damage and inflammation [5, 21], however, the literature is still unclear.

### Morphologic changes of the masticatory muscle and mandible

Unlike the skin and submandibular gland, the muscle and mandible demonstrated no visual external radiation-induced alterations upon dissection. As both muscle and bone tissue have low turnover rates and thus are less radiosensitive [4], this was not unexpected. However, the irradiated mandibles demonstrated a considerably paler coloration than the non-irradiated control mandibles following decalcification with 10 % EDTA for five weeks. As the histopathology of the irradiated mandible is understood today, progressive occlusion and obliteration of small vessels lead to reduced cell number, hypovascularity, fibrosis and fatty degeneration of the bone marrow [4, 5, 23]. Hypoplasia or aplasia of bone marrow is thus common after standard fractionated radiotherapy. The hematopoietic cells are decreased or absent and replaced by adipocytes. As a result, the bone marrow turns pale as the yellow-colored adipocytes replace the normal red hematopoietic marrow [24].

The H&E stained mandible sections demonstrated hyperkeratinization of oral gingival epithelium, dense connective fiber bundles and reduced number of fibroblasts in all connective tissues related to the tooth, and disturbed development of teeth under eruption. However, the crown teeth proved to be normal. A study by de Araujo et al. [25] supports these findings demonstrating that rats exposed to a single dose of 15 Gy presented statistical difference in tooth eruption rate from day six of the experiment [25]. Further, a study by Kaste et al. [26] reported adverse oral-dental sequelae among childhood cancer survivors treated with radiotherapy and chemotherapy. Radiotherapy was demonstrated to damage the tooth bud, thereby causing among other factors growth retardation of teeth, arrested root development, enamel hypoplasia, incomplete calcification and atrophy of underlying soft tissue [26]. This is consistent with our results verifying disturbed root development, dentin- and cementum formation, pulp necrosis and acute inflammation of unerupted teeth. Necrosis of the pulp is most likely an effect of the reduced vascularity observed in an area where good vascular supply is needed for tooth development. As there was a distinct difference between the radiation effect on teeth under eruption (adverse) and crown teeth (normal), the alterations in tooth development reflects the time point at which radiotherapy was initiated. The degree and severity of the radiation effects on dental health depend on the child’s age at diagnosis and the type and dose of radiation, with a low prevalence of defects in those children who had been treated after the amelogenesis of teeth was completed [26, 27].

In the present study the mandibular bone tissue was vital, as is the desired result in a patient situation. However, a reduced number of osteoblasts indicates a reduced capacity for growth and repair. The likelihood of mandibular necrosis by conventional fractionation is estimated at a 5 % incidence to 60 Gy [7].

### Vascularity

Damage to the vasculature following exposure to ionizing radiation has been recognized for a long time. The blood capillaries and sinusoids are the most radiosensitive, while the larger arteries having a muscular wall are less radiosensitive [24]. Consistently, the fine vasculature of the dermis, submandibular gland and muscle was compromised in the present study. The endothelial cells are only conditionally proliferating, and accordingly the vascular damage caused by irradiation heals poorly. As the vascular damage progresses, the initial increase in permeability gives way to decreased vascular perfusion resulting in ischemia and necrosis [21, 24]. In accordance with our results, vascular change leads to secondary tissue injury as a consequence of reduced perfusion.

### Collagen content

Fibrosis is a well-known consequence of radiation [21]. However, significantly increased fibrosis of the skin could not be verified in our animal model by analysis of transmission electron microscopy pictures. A tendency of increased fibril density in skin could, however, be obtained. This could be explained by the choice of area examined, as the collagen content was examined in the outer part of the radiation field and not the isocenter. One might assume the difference to be larger closer to the isocenter of radiation. The histologic examination clearly verified this assumption as the collagen network was dense and abundant in the dermis after radiotherapy. Additionally, a minor displacement in collagen fibril diameter towards larger fibrils following radiotherapy was demonstrated in the present study. It was further evident during dissection that the jaw region exposed to radiation generally had hard, tough tissue linings and extensive connective tissue growth distinctly dissimilar to the same region in control animals. As a result one can conclude that 5 × 15 Gy radiotherapy induced increased fibrosis, especially of the skin, in this animal model.

## Conclusion

The present study demonstrates histological changes attributed to radiotherapy of HNC in humans. The radiation-induced tissue injury can be reproduced in a rat model by external irradiation using a cumulative total dose of 75 Gy, fractioned in 15 Gy treatments every other week five times, with a post-radiation period of six weeks. Thus, we have established a rat model of radiation injury that can be used in future therapeutic evaluation studies.

## Abbreviations

EDTA: Ethylenediaminetetraacetic acid
Gy: Gray
H&E: Hematoxylin and eosin
HBO: Hyperbaric oxygen
HNC: Head and neck cancer
MU: Motor units
PBS: Phosphate buffered saline
PBS-TX: PBS with 0.3 % Trition® X-100
PFA: Paraformaldehyde
SD: Standard deviation
TBS: Tris buffered saline
